# Investigation into the Mechanism of Waterborne Polyurethane Modification of Ultrafine Talc and Its Impact on the Properties of Polypropylene Plastics

**DOI:** 10.3390/polym17010067

**Published:** 2024-12-30

**Authors:** Xianrong Yang, Huan Shuai, Gaoxiang Du, Jiao Wang, Jie Shen

**Affiliations:** 1 School of Materials Science and Technology, China University of Geosciences, Beijing 100083, China; 2103210011@email.cugb.edu.cn (X.Y.); shuaihuan@email.cugb.edu.cn (H.S.); 2 Beijing Yiyi Star Technology Co., Ltd., Beijing 100089, China; 3 School of Basic Education, Beijing Polytechnic College, Beijing 100042, China; wj@bgy.edu.cn (J.W.); sj@bgy.edu.cn (J.S.)

**Keywords:** talc, waterborne polyurethane, modifier, mechanical properties, polypropylene

## Abstract

In this study, waterborne polyurethane (WPU), a novel modifier, was used for the wet surface modification of talc, and its mechanism was investigated. Polypropylene (PP)-based composites with modified talc were synthesized and subjected to an examination of their mechanical properties. The wetting contact angle demonstrated that the modified talc exhibited an excellent modification effect at a specific amount of modifier (2.0 wt.%). The X-ray diffraction (XRD), Fourier-transform infrared (FTIR), and X-ray photoelectron spectroscopy (XPS) results indicated the successful coating of WPU on the surface of the talc particles. SEM images revealed that modified talc displayed improved wettability, compatibility, and dispersion in PP/talc + WPU composites. The mechanical properties results showed that the PP/talc + WPU composites ensured superior comprehensive properties with a flexural strength of 55.9 MPa, impact strength of 4.72 kJ/m^2^, tensile strength of 34.8 MPa, and elongation of breaks of 32.4%. The incorporation of WPU-modified talc into plastic materials has been synthesized to leverage its beneficial properties, leading to reduced production costs and improved performance and functionality of the final product.

## 1. Introduction

Talc is a unique, layered, non-metallic mineral [1,2], which is generally used as a filler in ceramics [3,4], greases [5], papers [6,7], plastics [8,9,10,11], coatings [12] and in other industries [13,14,15,16,17]. The application of talc powder in plastics has many advantages, owing to its unique microscopic sheet structure. This structural characteristic enables the talc powder to improve the rigidity, impact strength, flexural modulus, and thermal stability of plastic products.

In practical applications, the properties of polypropylene (PP)/talc composites are affected by the dispersion of talc in their matrix. Although talc has a better affinity for organic components than calcium carbonate and other fillers, there are broken ends of silicon ions and magnesium ions on the surface of ultrafine talc powder due to crushing. These sections have a strong hydroxyl bonding ability [18], and there are hydroxyl groups between the talc layers [19,20]. The hydrophilic property makes the formation of aggregates more simple when preparing PP/talc composites, which makes it difficult for talc to be uniformly dispersed in the polymer matrix, thus easily causing interface defects between the base material and the filler, and the direct application effect is not good. Therefore, it is necessary to carry out surface treatment of talc to enhance its compatibility with the organic matrix and enhance the mechanical properties of the composites. Recently, the demand for surface-modified talc products has increased.

In recent years, the surface modification of talc has gained widespread attention and significant progress worldwide. Common modifiers include silanes [21,22], titanates [23,24], and polymers, aimed at improving the affinity between talc and plastic substrates. However, there is still a need to develop new modifiers that offer better modification effects, lower costs, and more environmentally friendly options, which would further expand the functionalization and industrial application of talc. Although most studies focus on talc as a filler in polymer, typically being controlled at <10 wt.% [22], achieving higher filler content while ensuring good compatibility between talc and the polymer remains a challenge. Successfully increasing the filler content would not only reduce polymer usage but also enable high-value utilization of talc. Additionally, talc is commonly used as a modifying filler in polyurethane foams [18,25], demonstrating good compatibility between talc and polyurethane. However, research on the modification of talc by polyurethane is still scarce, presenting a potential area for further exploration and development.

In this study, the classical wet modification process was improved upon. Waterborne polyurethane (WPU) was used to modify ultrafine talc powder, which was then employed as an additive in PP plastics. The impact of talc addition and its modification on the final properties of PP-based composites was assessed. Tests, including regarding oil absorption, contact angle, and sedimentation time, were conducted to evaluate the surface properties of the talc before and after modification. The effect of the modification process on the affinity of talc in the organic phase was verified through ethanol dispersion and characterized using scanning electron microscopy (SEM). X-ray diffraction (XRD), Fourier-transform infrared spectroscopy (FTIR), and X-ray photoelectron spectroscopy (XPS) were used to confirm the modification effect and analyze the modification mechanism. Mechanical properties of PP-based composites were tested and the dispersion of both two kinds of talc in the plastic matrix were further examined using SEM.

## 2. Experiment

### 2.1. Materials

Ultrafine talc(6650A) was used as a raw material, provided by Liaoning Runhai Material Co., Ltd. (Anshan, China). The waterborne polyurethane monomer (WPU, NKWPU 10502), supplied by Guangdong Huashi New Material Technology Co., Ltd. (Guangzhou, China), is a white emulsion with an effective ingredient content of 31 wt.%. It consists of 1,4-butanediol and a hydrophilic diisocyanate, with the molecular weight ratio of the two monomers being 1:1. Dibutyl phthalate (DBP) was provided by Aladdin Biochemical Technology Co., Ltd. (Shanghai, China). Erucic acid amide (cis-13-docosenoic acid amide) (EAA) was provided by Shanyi Plastic Chemical Co., Ltd. (Dongguan, China). Distilled water was prepared in the laboratory (resistivity ≥ 18.0 MΩ.cm).

### 2.2. Modification of Talc Particles

The modification of talc was carried out using an improved wet process. Talc particles (100.0 g) and distilled water (400.0 g) were placed in a beaker with a certain dosage of WPU (0.50 wt.%, 1.00 wt.%, 1.50 wt.%, 2.00 wt.% and 2.50 wt.% of talc mass) after talc and water were mixed well by stirring, and were then kept stirring and while being heated to 90 °C. The mixture was stirred at 900 rpm at 90 °C for 60 min. The mixture was then cooled to room temperature, filtered, and dried at 90 °C. The dried samples were dispersed and packed in bags for subsequent use.

### 2.3. Preparation of PP-Based Composites

**Figure 1:**
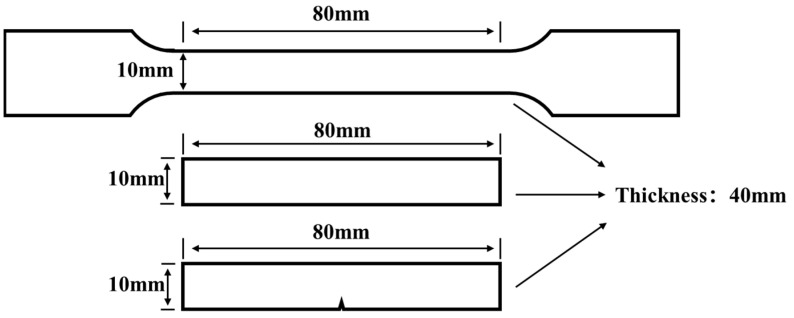
The dimensions of PP-based composite injection molding splines; top-down: tensile test spline, flexural test spline, and impact test spline.

Composite preparation is divided into mixing, extrusion, and injection molding processes. (1) The mixing process was performed using a high-speed mixer, provided by Tongsha Plastic Machinery Co., Ltd. (Zhangjiagang, China). The specified ratios of PP plastic, talc powder (modified with 2.0 wt.% of WPU or unmodified), and EAA were mixed as shown in Table 1. They were then stirred at 500 rpm for 5 min, then the mixture was bagged for later use. (2) The extrusion process was carried out using a NE27E/40-1500 single-screw extruder provided by Zhongzhuang Co., Ltd. (Chengdu, China). The extrusion temperature was set to 210 °C. After extrusion, the melted mixture was cooled and solidified directly through the water bridge and then dried and bagged after granulation using a pelletizer. (3) The injection molding process was performed using a YJ79-200 hydraulic injection molding machine provided by Jiangdong Machinery Co., Ltd. (Chongqing, China). The melting temperature of the machine was set to 210 °C. The granulated particles were placed into a hydraulic injection molding machine to be heated, melted, and injected with a pressure of 5 MPa into the mold to form splines, as shown in Figure 1.

### 2.4. Characterizations

The oil absorption value was determined as follows. The sample powder (1.000 g ± 0.0005 g) was weighed and placed on a clean glass plate (20 cm × 20 cm). DBP was filled in an acid burette with an accurate grade of grade A. DBP was slowly added dropwise to the samples. Simultaneously, the sample and DBP were scraped back and forth using a scraper for mixing. The sample was bonded to DBP to form an agglomerated and free dry sample, which was regarded as the endpoint.

A_0_ = V/M

A_0_: oil absorption value (mL/g); V: volume of DBP consumed (mL); M: mass of the sample (g).

Settling time in water: The purpose of the modification was to improve the dispersion of talc in the organic phase. A shorter settling time indicates better dispersion stability of talc in the organic phase. During the test, talc (0.250 g ± 0.0005 g) and water (50 mL) were placed in a beaker and stirred for 10 min using a magnetic stirrer, and then 25 mL of the suspension was placed in a measuring cylinder and a timer was started. When the 5.0 mL scale line could be seen, the time indicated was noted as the settling time.

Contact angle: The measurement was performed using a KRUSS DSA100 contact angle measuring instrument provided by Krüss Scientific Instruments Inc., (Hamburg, Germany). A powder sample (5.0 g) was pressed flat using a tablet press. The sessile drop method was used, and the water droplet size used was 2 μL. The contact angle was calculated by Young’s Laplace method.

SEM: (1) The sample was added to an anhydrous ethanol solution at a mass fraction of 0.05%, and an ultrasonic probe provided by Shenxi Ultrasonic Instrument Co., Ltd. (Shanghai, China) was used for ultrasonic treatment for 1 min. A total of 10 μL of the liquid was dropped on the silicon wafer using a pipette. The gold sputter coating was performed at a working current of 20 mA for 120 s. (2) The talc/PP composites with talc addition of 5 wt.% were immersed in liquid nitrogen for 5 min, and then the samples were taken out and checked for brittle fracture, and then sputtered with gold sputter coating at a working current of 20 mA for 120 s. (3) The surface morphologies of talc, modified talc, and talc/PP composites after brittle fracture were observed using a Hitachi Electronics JSM-7610F, provided by JEOL Ltd. (Tokyo, Japan)

XRD: An Ultima IV X-ray diffractometer Rigaku Corporation (Tokyo, Japan) was used to test and analyze the crystal structure of talc before and after modification. The scanning speed was 10°/min, and the 2θ angle range was 5–90°.

FTIR: A Bruker ALPHA FTIR spectrometer provided by Bruker Corporation (Billerica, MA, USA) was used to characterize talc before and after modification, and the wave number range was 4000–400 cm^−1^. Before conducting the sample test, an air background test was performed, recording at least 32 scans to obtain a stable background spectrum. For the test, a small amount of powder or liquid sample is placed in the light path and at least 32 scans are recorded, during the measurement. The previously recorded air background spectrum was subtracted from the sample spectrum to obtain the net sample spectrum.

Particle size distribution: The particle size distribution of talc was tested using a BT-2600 laser particle size distributor, which was provided by Baite Instrument Co., Ltd. (Dandong, China): (1) The equipment was preheated for 30 min, and the refractive index and absorbance parameters were set as the standard parameters of talc in the test software. (2) A small amount of the uniformly mixed sample (0.250 g) was dispersed in 100 mL of water and a small amount of sodium hexametaphosphate was added as a dispersant. The probe was ultrasonically treated for 5 min at 300 W power to prevent particle agglomeration. (3) An ultrasonically dispersed sample is added to the device for testing. After the data were stabilized, three sets of data were automatically measured and averaged. (4) The laser particle size distribution instrument was automatically cleaned.

Tensile test, impact, and flexural test: An ETM 304C universal testing machine provided by Wance Experimental Equipment Co., Ltd. (Shenzhen, China) was used for testing. PP-based composites were used for each mechanical performance test. During the tensile test, the fixture spacing was 110 mm and the test speed was 20 mm/min. In the flexural test, the gap between the supports was 60 mm and the test speed was 5 mm/min. The Charpy impact test used a KY-JZL5D simply supported beam impact testing machine provided by Keyue Instrument Co., Ltd. (Hefei, China). During the test, the zero point was reset to a fixed position, the test energy was 7.5 J, and the impact speed was 3.8 m/s. All mechanical performance tests were repeated 3 times.

## 3. Results and Discussion

### 3.1. Characterization of Talc Particles

#### 3.1.1. Performance Index Test of Talc Particles

**Figure 2:**
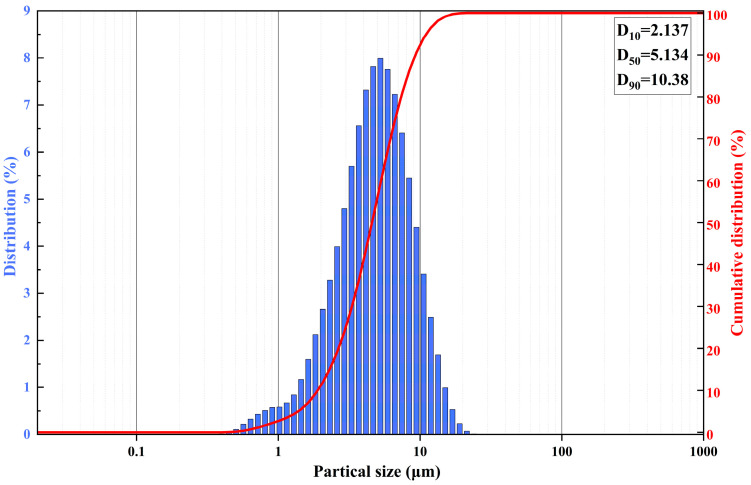
Particle size distribution of ultrafine talc.

The particle size of talc is shown in Figure 2 and was obtained using a laser particle size distributor.

**Figure 3:**
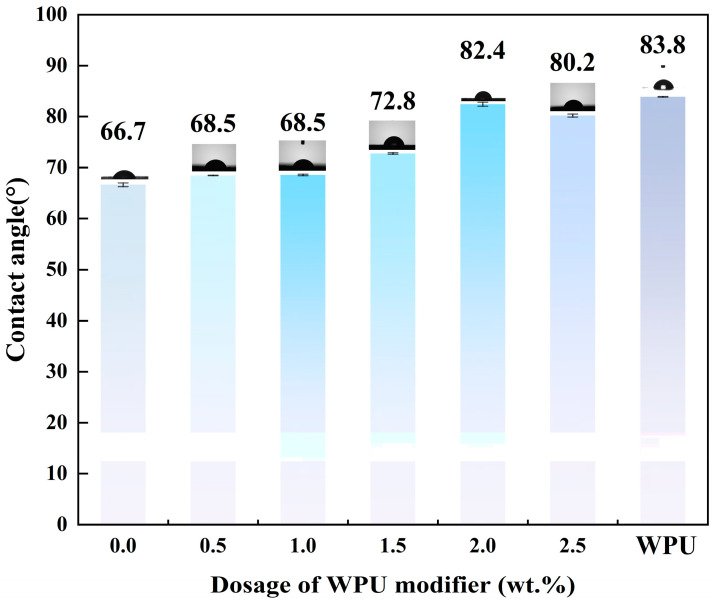
Wetting contact angle of talc, talc modified with different dosages of waterborne polyurethane (WPU), and dried WPU.

The talc before and after modification was evaluated by the sessile drop method, and the results are shown in Figure 3. The wetting contact angle of the unmodified talc was 66.7°, indicating the property of hydrophilicity. As the modifier dosage increased, the contact angle gradually increased. During the modification process, when the WPU content reached 2.0 wt.%, the contact angle increased to 82.4°, which is close to the contact angle of dried WPU. This indicates that WPU successfully coated the surface of the talc particles, allowing the powder to effectively integrate with the PP plastic system. Upon increasing the dosage of WPU during the modification process, the contact angle was significantly reduced. This phenomenon can be further explained as follows: excessive WPU undergoes polymerization reactions in water, resulting in the formation of a multilayer of WPU coatings on the talc surface. Within this structure, the hydrophobic groups of the inner layer of the WPU are oriented outwards, while the hydrophilic layer of the outer layer of the WPU is bound to the outwards faces. This unique layered structure gives the talc modified with 2.5 wt.% WPU higher hydrophilicity, leading to a reduction in its contact angle. It is evident from the trend in the contact angle changes that modification with WPU plays a significant role in enhancing the hydrophilicity of talc.

**Figure 4:**
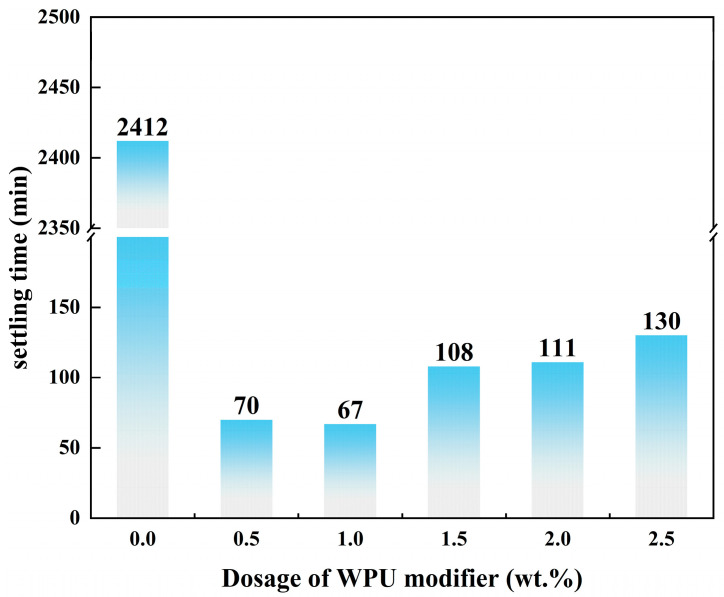
Settling time of talc and modified talc with different dosages of WPU in water.

The primary purpose of the modification is to improve the dispersion of talc in the organic phase, which can be evaluated by the settling time of the modified and unmodified talc in water. The results of the settling-time tests are shown in Figure 4. The settling time of the unmodified talc in water was more than 2400 min, which indicates that talc was well dispersed after bare magnetic stirring. As the dosage of the WPU modifier was increased during modification, the time required for the modified talc to settle in water also increased. The shortest time required for sedimentation was for talc modified by 1.0 wt.% WPU modifier and the settling time was 67 min.

**Figure 5:**
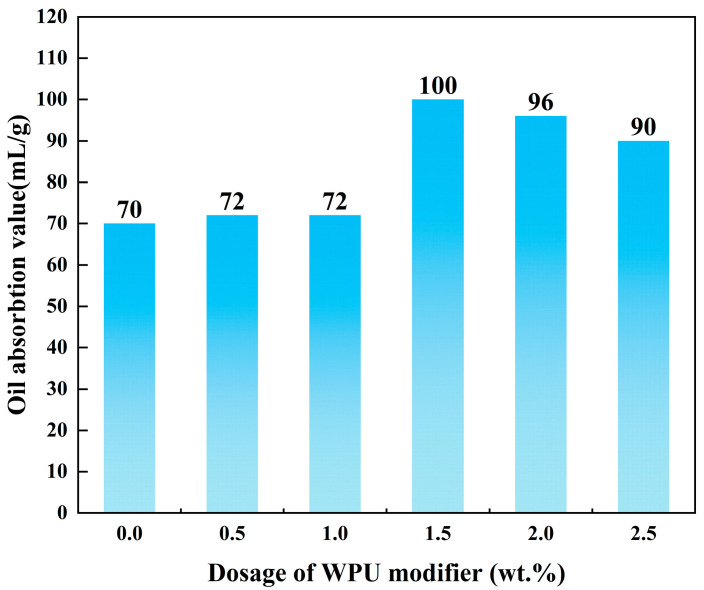
Oil absorption value of talc and modified talc with different dosages of WPU.

The oil absorption value is one of the most important characteristics for evaluating the modification effects of inorganic powders. The results of the oil absorption tests are shown in Figure 5. When the powders were applied to the polymer composite material system, the lower the oil absorption was, the easier the mixing with the plastic system was. The oil absorption value of the unmodified talc powder was 70 mL/g, whereas that of the modified talc increased. Since the modifier was added more than 1.0 wt.%, the oil absorption value did not change significantly. The oil absorption value of talc modified with 2.5 wt.% WPU was the maximum value of 130 mL/g. The results of the settling time and oil absorption values were not consistent with the measurement results of the contact angle. This is because the particle size of the talc powder changed after the modification. The contact angle results provided the most accurate reflection of the affinity between talc and the organic phase. Therefore, it is believed that the modification effect of talc powder is the best with a modification condition of 2.0 wt.% WPU dosage, and this dosage was used to evaluate the formulation of PP/talc + WPU composites.

#### 3.1.2. Modification Mechanism of Talc

**Figure 6:**
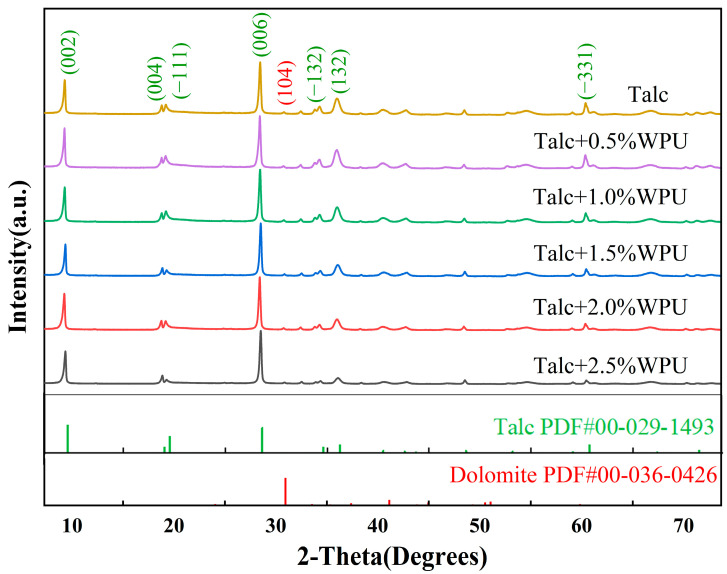
XRD patterns of talc, modified talc, and standard PDF cards of talc and dolomite.

To determine the effect of the modification process on the crystal structure of talc, XRD measurements were performed for unmodified and modified talc. The results are shown in Figure 6. The diffraction peaks at 2θ angles of 9.49°, 18.99°, 19.50°, 28.59°, 34.58°, 36.18°, and 60.72° corresponded to the (002), (004), ($\overset{-}{1}$11), (006), ($\overset{-}{1}$32), (132), and (331) crystal faces of talc. At a 2θ angle of 30.94°, a subtle diffraction peak was observed, which corresponds to the (104) crystal plane of dolomite [26]. This finding suggests that the talc powder contained a small amount of dolomite impurities, which was further corroborated by subsequent XPS analysis. The results also show that raw talc has a very significant crystal structure when the median diameter is approximately 5 μm, and the diffraction peak position of each crystal plane can accurately correspond to the diffraction peak position recorded in PDF#00-029-1493 [27]. The crystal structure of talc modified with different amounts of WPU did not change, and the position of the diffraction peak also accurately corresponded to the unmodified talc and its standard card. This shows that the modification process did not change the crystal structure of talc. This phenomenon has been mentioned in the same surface-modification work [28]. Comparing the diffraction peaks corresponding to the (006) crystal face, the intensity of the diffraction peak of talc modified with 2.0 wt.% WPU was the lowest. It has been shown that the diffraction peak intensity of minerals modified by organic components decreases under the same conditions. This result can be explained by the successful coating of the surfaces of the particles with the WPU modifier.

**Figure 7:**
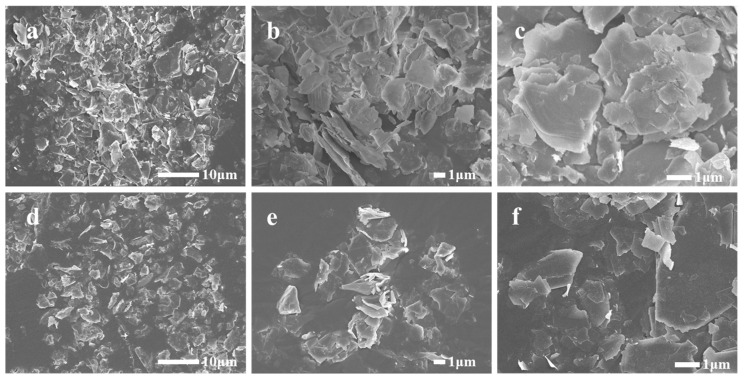
SEM images of talc (**a**–**c**) and talc modified by 2.0 wt.% of WPU (**d**–**f**).

The SEM images of talc before and after modification are shown in Figure 7. It is evident that the particle size of talc primarily ranges from 1 to 10 μm, with a minority of particles attaining sizes below 1 μm, which aligns with the particle size distribution results obtained through laser particle size analysis. It can also be seen that the talc particles crystallize well, and most of the particles had a flaky structure. Because of the relatively small particle size distribution, the agglomeration of unmodified talc particles was more obvious, and no dispersed single particles were observed. Conversely, the dispersion of the modified talc particles is significantly improved, and a small number of particles can achieve single-particle dispersion. Even if some particles formed agglomerates, the size of these agglomerates was less than 5 μm. The surfaces of these particles did not exhibit the presence of a WPU modifier. Furthermore, upon gold plating of the modified samples, a notable discharge phenomenon was still observed in the electron microscopy images. This observation can be attributed to the cleavage and destruction of the WPU modifier on the particle surface which underwent bombardment with high-energy electron beams. This situation causes not only the deterioration of the altered talc surface modifier, but also the degradation of the gold sputter coat, ultimately leading to a considerable discharge phenomenon.

**Figure 8:**
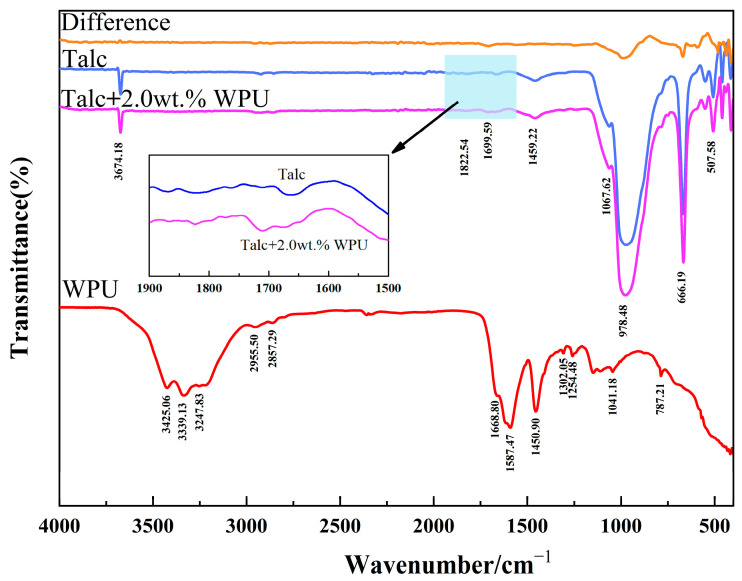
FTIR spectra of talc, talc modified by 2.0 wt.% of WPU, WPU, and spectra difference for two kinds of talc.

The FTIR spectra of the modified and unmodified talc samples are shown in Figure 8. In the infrared spectrum of talc, the absorption peak observed at 3674 cm^−1^ is attributed to the symmetric stretching vibration of the H-O bond, which corresponds to the free hydroxyl group present in talc. Meanwhile, the peak at 978 cm^−1^ arises from the Si-O stretching vibration within the silicon–oxygen tetrahedron. Additionally, the absorption peak recorded at 666 cm^−1^ represents Mg-O vibration absorption in the magnesium oxygen octahedron [5,12,25].

In the absorption spectrum of WPU, the peaks at 2955 cm^−1^ and 2857 cm^−1^ corresponded to the stretching vibration of the C-H bond. The peak at 3425 cm^−1^ represents the symmetric stretching vibration of the N-H bond, while the peak at 3339 cm^−1^ corresponds to the antisymmetric stretching vibration of the N-H bond. Furthermore, the N-H stretching vibration occurred at 3247 cm^−1^, while the in-plane bending vibration of N-H was observed at 1587 cm^−1^. Additionally, C=O stretching vibration was observed at 1668 cm^−1^. The peaks at 1254 cm^−1^ and 1302 cm^−1^ correspond to the extension of the C-N bond. The stretching vibration of the C-O bond was detected at 1041 cm^−1^, and the out-of-plane bending vibration of the N-H bond was located at 787 cm^−1^ [18].

As shown in Figure 8, The “difference “curve represents the signal difference between talc before and after modification, which was obtained by subtracting the FTIR spectrum of unmodified talc from that of the modified talc. When comparing the spectra, the addition of WPU-modified talc introduces a new absorption peak near 1700 cm^−1^, which aligns with the absorption peak resulting from the C=O stretching vibration and the in-plane bending vibration of N-H in WPU. This indicates that the modification successfully triggered a condensation reaction between the isocyanate and polyols in WPU and the hydroxyl groups on the talc surface, resulting in the formation of long polyurethane chains on the surface. Additionally, the emergence of a subtle absorption peak between 1200 and 1300 cm^−1^ corresponds to the stretching vibration of the C-N bond, further demonstrating the effectiveness of the modification.

**Figure 9:**
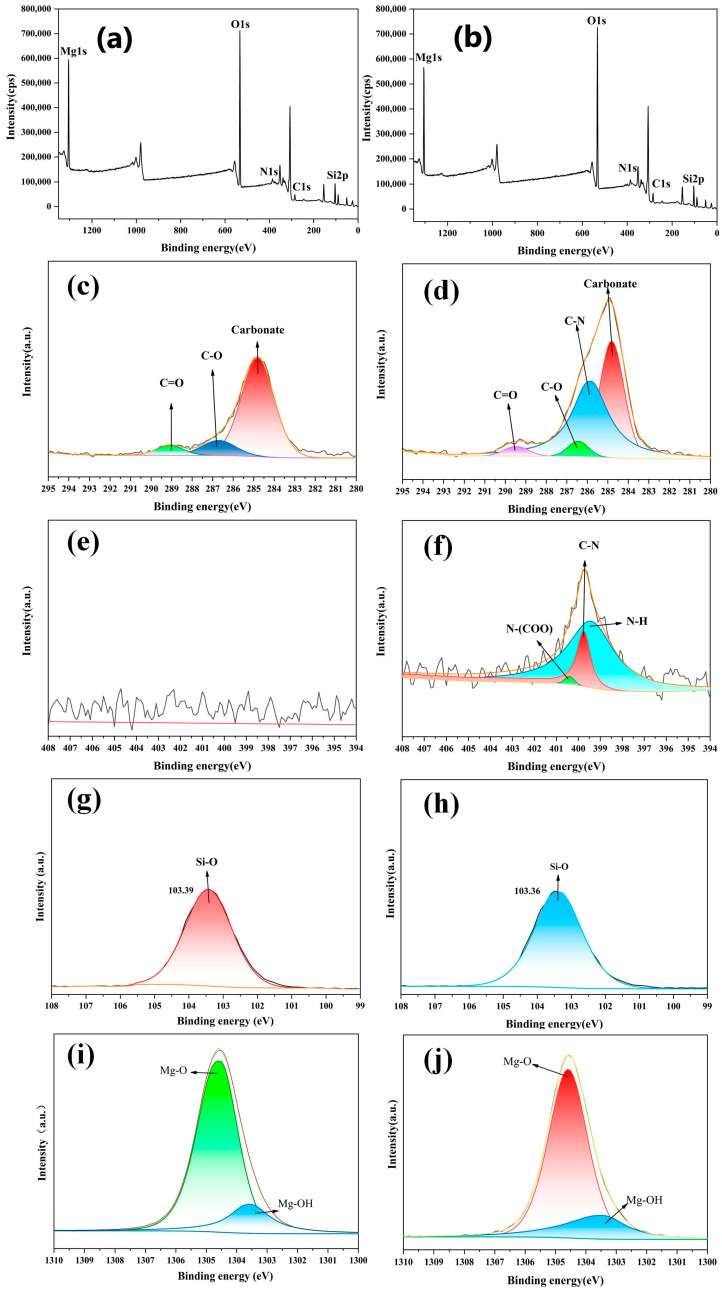
XPS spectra of talc (**a**,**c**,**e**,**g**,**i**) and talc modified by 2.0 wt.% of WPU (**b**,**d**,**f**,**h**,**j**).

Figure 9 shows the XPS spectra of the modified and unmodified talc. As shown in Figure 9a,b, it can be observed that the intensity of the C1s peak near 285 eV for the modified talc is significantly higher than that of the unmodified talc. This is because the attachment of modifiers introduces additional C-N bonds on the surface of talc and increases the number of C-O and C-H bonds.

In Figure 9c, the peaks at 289.13 eV, 286.71 eV, and 284.80 eV correspond to C-O, C=O bonds, and C-C bonds, respectively. These chemical bonds originate from the small amount of dolomite impurities present in the talc. The presence of dolomite is consistent with previous XRD results. Similarly, in Figure 9d, binding energy peaks corresponding to C-O, C=O, and C-C bonds were also observed at 289.41 eV, 286.46 eV, and 284.80 eV. The peak at 285.84 eV corresponds to the C-N bond, which is a unique chemical bond present on the surface of the modified talc. This indicated that the modification step successfully introduced C-N bonds to the surface of the talc particles, and the modifier was successfully incorporated into the talc particles.

Figure 9e,f shows the spectral peaks of N1s. Talc does not contain nitrogen. The appearance of nitrogen on the surface of the modified talc led to the emergence of spectral peaks in the XPS results. Among them, the binding energy of 399.75 eV corresponds to the C-N bond, 400.38 eV corresponds to the N-(COO) bond and 399.41 eV corresponds to the N-H bond. The emergence of distinct spectral peaks in the XPS results of the modified talc indicated that the modifiers were attached to the surface of the talc particles. Figure 9g,h shows the spectral peaks of Si2p. The positions and heights of the binding energy peaks for the Si-O bond on the surface of talc and modified talc are similar, with binding energy peak positions of 103.39 eV and 103.36 eV, respectively. This indicates that the modifier did not directly interact with the silica tetrahedra. Figure 9i,j shows the spectral peaks of the Mg2s. In Figure 9i, the peaks at 1304.55 eV and 1303.55 eV correspond to Mg-O and Mg-OH bonds, respectively, which originate from the magnesium–oxygen octahedra and the hydroxyl groups associated with magnesium at the terminal surface [15].

**Figure 10:**
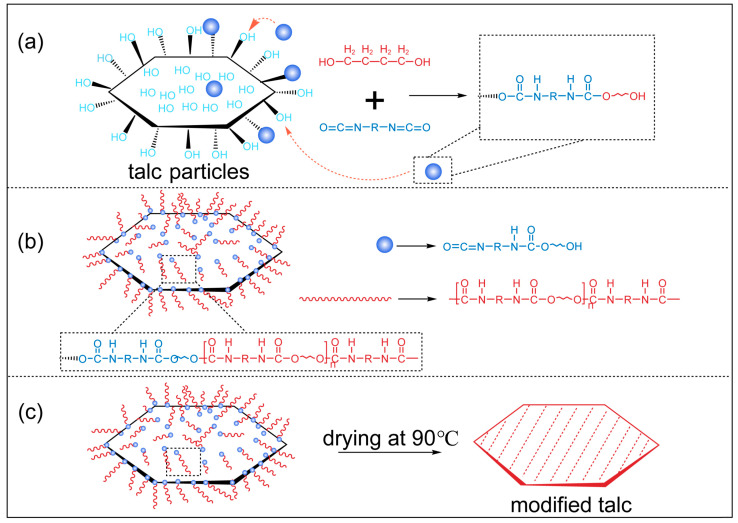
Mechanism of WPU modified talc. (**a**) Isocyanate is grafted onto the surface of talc powder. (**b**) Diol and isocyanate continue to condense to form polyurethane chains. (**c**) During the drying process, the polyurethane chains are cross-linked to form a coating structure on the surface of the talc powder.

Based on the aforementioned characterization analysis, a mechanism for the modification of talc using the WPU modifier was proposed, as depicted in Figure 10. The talc surface features interlayer hydroxyl groups and free hydroxyl groups on its terminal faces. When thoroughly mixed and heated to a certain temperature, these hydroxyl groups can undergo condensation reactions with the isocyanates and diols present in the WPU. After the isocyanates were grafted onto the hydroxyl groups on the talc surface, the remaining diols and isocyanates continued to graft and extend the chain on the surface and on the already condensed amine ester groups. During the drying process, the polyurethane long chains on the talc surface further crosslinked and formed a microbubble coating layer on the talc surface, achieving the desired surface modification effect. The alkyl-bearing R groups reduce the hydrophilicity of the talc surface. Such modifications enable better dispersion and encapsulation of talc in organic phases, as demonstrated by its application as a filler for PP.

### 3.2. Characterization of PP-Based Composites

**Figure 11:**
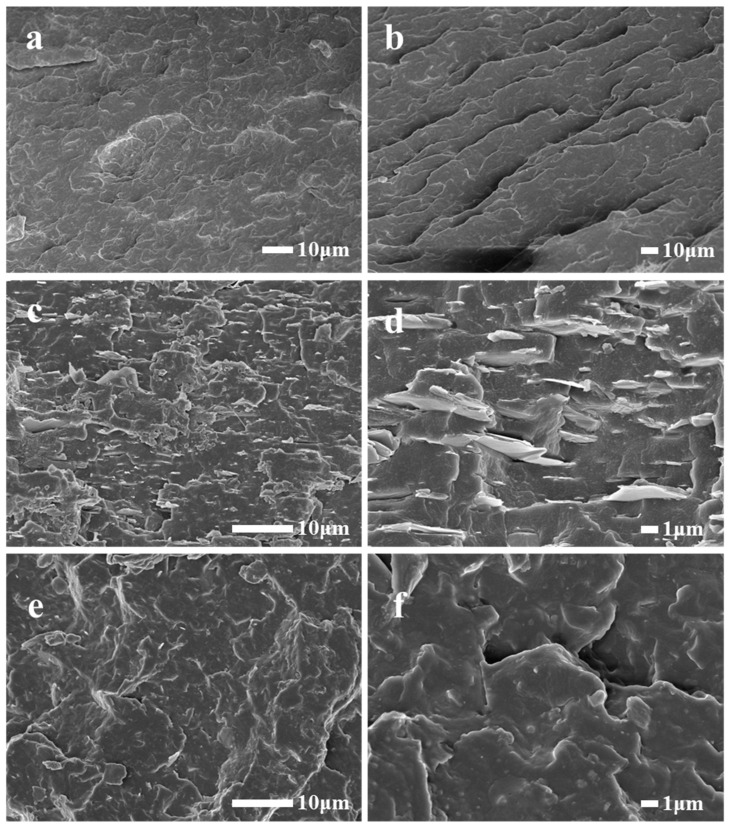
SEM images of Polypropylene (PP) (**a**,**b**), PP/5% talc composites (**c**,**d**), and PP/5% talc + WPU composites (**e**,**f**).

Figure 11 presents the electron microscopy images of freshly fractured surfaces obtained through a cryogenic brittle fracture in liquid nitrogen for PP, PP/5% talc composite, and PP/5% talc + WPU composite. The fractured surface of pure PP observed in Figure 11a,b is relatively neat, with the microfracture direction of the PP plastic running parallel to the observation surface. Significant differences were observed in the electron microscopy images of the composites prepared with unmodified and modified talc. In the PP/5% talc composite, some parts of the microfracture are perpendicular to the observation surface, and very smooth planes can be seen on the vertical fractures, which are all flaky talc particles. In Figure 11d, large gaps are observed between the flaky talc particles and the PP matrix, indicating poor compatibility between talc and the PP matrix. In Figure 11c, it is evident that the talc particles are significantly agglomerated, suggesting poor dispersion of talc in the composite material. The agglomeration of talc particles and the gaps between talc and the PP matrix led to reduced strength in the local areas of the composite, making it susceptible to fracturing under stress. Similar phenomena have been reported in other talc/polymer composites [10,22,24]. In some studies, talc was treated with silane or phthalate-based modifiers and then incorporated as a filler into polymers. However, SEM images of these composites revealed that visible gaps between the talc particles and the polymer matrix still remained, indicating that while the affinity was improved, the interfacial bonding was not entirely resolved.

Figure 11e,f shows the PP/5% talc + WPU composite. In the SEM images, the fracture surfaces were parallel to the observation plane, and almost no directly exposed flaky talc was visible. This indicates that the compatibility and wettability between the modified talc particles and PP matrix were particularly good in this composite. The talc layers were completely encapsulated in the PP matrix. There was also no visible agglomeration of the talc particles, suggesting that the modification process also improved the dispersion of talc in the PP matrix. The jagged edges of the fracture surface in Figure 11f are due to the reinforcing effect of encapsulated talc when a sample is under stress. The fracture surface must avoid talc particles, which results in an uneven appearance. It is evident from Fig. 11 that the modified talc exhibits superior affinity, wettability, and dispersion in PP plastic compared to what is typically observed in similar studies [10,22,23,29]. After modification, there are no visible gaps between the modified talc and the PP matrix. Such results strongly demonstrate the effectiveness of the modification by WPU.

**Figure 12:**
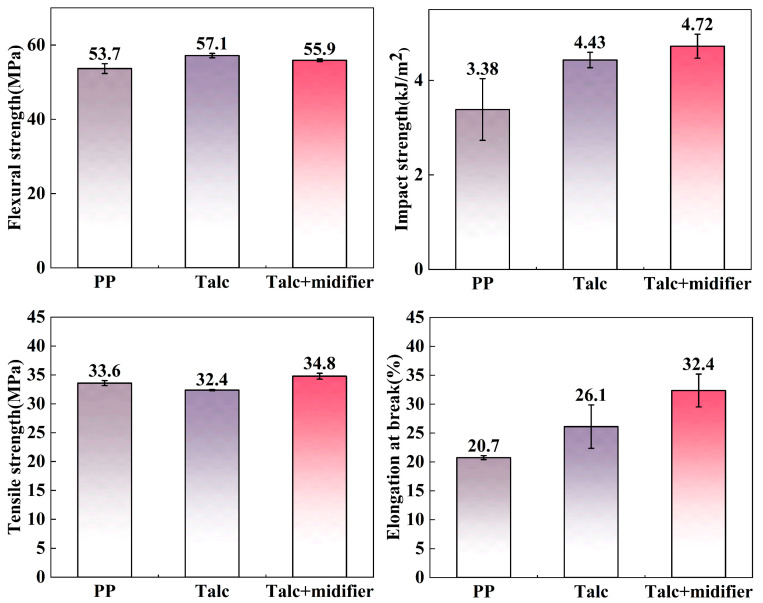
Impact, flexural, tensile strength, and elongation at break of pure PP, PP/20% talc composites, and PP/20% talc + WPU composites.

It can be seen in Figure 12 that the flexural strength, impact strength, tensile strength, and elongation at the break of the pure PP plastic sample were 53.7 MPa, 3.38 kJ/m^2^, 33.6 MPa, and 20.7%, respectively. In contrast, the flexural strength, impact strength, tensile strength, and elongation at the break of the PP/20% talc composite were 57.1 MPa, 4.43 kJ/m^2^, 33.6 MPa, and 26.1%, respectively. The PP/20% talc + WPU composite exhibited a flexural strength of 55.9 MPa, impact strength of 4.72 kJ/m^2^, tensile strength of 34.8 MPa, and elongation at the break of 32.4%. Compared with pure PP, the tensile strength of the PP/20% talc composite samples was slightly reduced. This is due to insufficient tight binding between talc and PP plastic, leading to stress concentration at defects under stress conditions, thereby causing a decrease in strength. However, the tensile strength of the PP/20% talc + WPU composite was slightly higher than that of pure PP, which can be attributed to the excellent compatibility and wettability between the modified talc and the PP matrix. The elongation at break, impact strength, and flexural strength of PP/20% talc composite are superior to those of pure PP plastic. This advantage can be attributed to the layered structure of talc, which provides strength when subjected to impact and flexural stresses. Under tensile stress, the talc layers aligned parallel to the direction of tension could bear the tensile force, and enabled the sample to elongate evenly. The modified talc, owing to its improved interfacial interaction with the PP matrix, exhibited higher impact strength and elongation at the break. However, these may not provide sufficient strength when subjected to flexural stress. The results of this study align with existing research on talc modification and its application in plastics, while also revealing some differences. Talc is widely used as a filler in PP composites, where moderate amounts improve mechanical properties like flexural and impact strength [9,30,31]. However, other studies have primarily focused on talc contents below 10%, where the mechanical performance of the composites is generally improved, as higher talc concentrations may lead to poor interfacial bonding and stress concentration [10,29,30]. In this study, waterborne WPU modification significantly enhanced the impact strength and elongation at break of the composite, with improved compatibility between modified talc and the PP matrix. The talc content reached 20 wt.%, and although its layered structure helps resist impact and tensile stress, it does not effectively enhance bending strength under flexural stress.

## 4. Conclusions

A novel modifier, WPU, was utilized to achieve wet modification of talc, leading to a marked enhancement in the hydrophobicity of talc. This improvement was supported by the test results obtained for the wetting contact angle, oil absorption value, and sedimentation time. Furthermore, the dispersibility of modified talc in organic solvents was significantly improved. The dispersibility of the modified talc in organic solvents was significantly improved, as confirmed by SEM characterization. Through systematic studies, optimal results were achieved with 2.0 wt.% WPU content. XRD, FTIR, XPS, and SEM analyses further confirmed that WPU was successfully coated onto the talc particles.

The results of mechanical performance testing indicated that PP/20% talc + WPU composite material exhibited excellent overall performance. Its bending strength reaches 55.9 MPa, its impact strength measures 4.72 kJ/m^2^, its tensile strength stands at 34.8 MPa, and the elongation at break is 32.4%. Compared with the PP/20% talc composite, the use of modified talc as a filler for PP plastics enhances the tensile strength and elongation at break of the plastic. Additionally, the impact strength of the plastic is improved by 39.6% compared to pure PP plastic. In summary, WPU could effectively hydrophobically modify talc at relatively low modifier dosages. Employing modified talc as a filler for PP plastics can reduce the usage of PP raw materials, lower the manufacturing cost of plastic, and improve its application performance. Consequently, WPU modifiers offer enhanced application prospects. The use of WPU modifiers has significant potential for enhancing the application prospects of talc in the plastic industry, making it a promising candidate for organic matrix-filled modifiers.

## Figures and Tables

**Table 1:** Samples and corresponding descriptions.

Sample	PP (wt.%)	Talc (wt.%)	EAA (wt.%)	WPU on Talc (%)
PP	100	0	0	0
PP/5% talc	94.6	5	0.4	0
PP/5% talc + WPU	94.6	5	0.4	0
PP/20% talc	79.6	20	0.4	2.0
PP/20% talc + WPU	79.6	20	0.4	2.0

## Data Availability

Data will be made available on request.
